# Soft template-assisted copper-doped sodium dititanate nanosheet/graphene oxide heterostructure for photoreduction of carbon dioxide to liquid fuels†

**DOI:** 10.1039/d2ra04283e

**Published:** 2022-08-26

**Authors:** Napat Lertthanaphol, Natthanicha Prawiset, Pornpinun Soontornapaluk, Nutkamol Kitjanukit, Wannisa Neamsung, Natpichan Pienutsa, Kittapas Chusri, Thirawit Sornsuchat, Prowpatchara Chanthara, Poomiwat Phadungbut, Panpailin Seeharaj, Pattaraporn Kim-Lohsoontorn, Sira Srinives

**Affiliations:** Nanocomposite Engineering Laboratory (NanoCEN), Department of Chemical Engineering, Faculty of Engineering, Mahidol University Nakorn Pathom 73170 Thailand Sira.sri@mahidol.edu; Advanced Materials Research Unit, Department of Chemistry, Faculty of Science, King Mongkut's Institute of Technology Ladkrabang Bangkok 10520 Thailand; Center of Excellence on Catalysis and Catalytic Reaction Engineering, Department of Chemical Engineering, Faculty of Engineering, Chulalongkorn University Bangkok 10330 Thailand

## Abstract

Photoreduction of CO_2_ to a high-value product is an interesting approach that not only captures CO_2_ but also converts it into other products that can be sold or used in industry. The mechanism for the CO_2_ conversion relies strongly on photo-generated electrons that further couple with CO_2_ and form active radicals for the reaction. In this research, we synthesized a heterostructure of copper-doped sodium dititanate nanosheets and graphene oxide (CTGN) following a one-step hydrothermal process with assistance from a sodium hydroxide soft template. The role of the template here is to facilitate the formation of the nanosheets, creating the nanosheet–graphene 2D–2D heterostructure. The heterostructure yields excellent charge mobility and a low charge recombination rate, while the nanosheet–graphene interfaces house active radicals and stabilize intermediates. The CTGN exhibits an outstanding photoactivity in the photoreduction of CO_2_, producing liquid fuels, including acetone, methanol, ethanol and i-propanol.

## Abstract 2

The copper-doped sodium dititanate nanosheets/graphene oxide heterostructure (CTGN) was synthesized following a one-step hydrothermal process, exhibiting an outstanding photoactivity in converting CO_2_ to acetone, methanol, ethanol and i-propanol.

## Graphical abstract

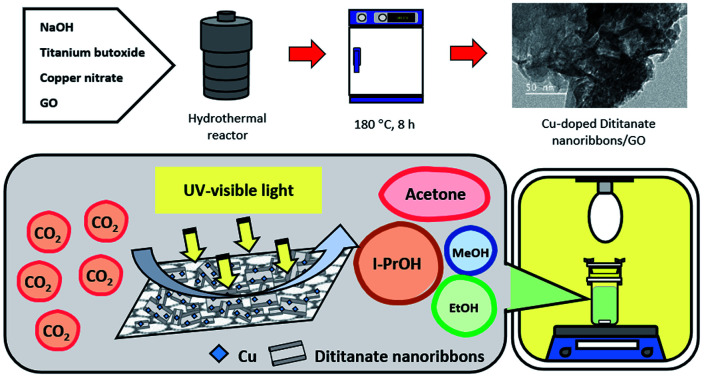

## 1. Introduction

Carbon dioxide (CO_2_) emission has become a topical issue due to global policies and strategic goals for a low carbon economy. The policies and goals are the main driving forces for the development of technologies for carbon capture and storage (CCS). Conventional approaches for CO_2_ management utilize a liquid or solid base for CO_2_ capture. The capture process is usually followed by a regeneration of the liquid or solid base, in which a significant amount of energy is consumed and the CO_2_ is released back into the atmosphere. The other approach is CO_2_ sequestration, involving the injection and storage of CO_2_ deeply in an underground site. The sequestration promises a dramatic reduction in CO_2_ emissions but is inhibited by the risk of CO_2_ leakage and requirements for long-term inspection. One alternative to CCS is CO_2_ conversion to a high-value product. The approach concerns not only the removal of CO_2_ but also the generation of commodities, such as methane,^1–3^ carbon monoxide,^2,3^ formaldehyde,^4^ methanol,^4,5^ and ethanol.^6,7^

Different techniques were introduced and demonstrated for CO_2_ conversion, including CO_2_ fixation and conversion by microalgae, CO_2_ hydrogenation by metal oxide catalyst,^8^ and CO_2_ splitting using a metal oxide electrocatalyst.^9^ Photoreduction of CO_2_ to liquid fuels is an attractive alternative that relies on photocatalysts such as zinc oxide (ZnO),^3,10^ cadmium sulfide (CdS),^2^ and titanium dioxide (TiO_2_).^5,6,10–19^ TiO_2_ is a popular photocatalyst that has been used in the decomposition of organic pollutants in wastewater. It has also featured heavily as a potent photocatalyst for CO_2_ conversion. An issue regarding TiO_2_ concerns the wide bandgap energy, which limits the number of photo-generated electrons resulting in a fast pairing rate of electrons and holes. TiO_2_ also responds only to UV light, preventing it from utilizing the full intensity of natural sunlight.

A photocatalytic heterostructure between two 2-dimensional nanostructures, defined as a 2D–2D heterostructure, can be an ideal photocatalytic platform that provides excellent charge mobility and charge separation.^13,15,20–22^ The heterostructure contains interfaces between two semiconductors with unequal bandgap values, which induces a local electric field that directs the flow of charge carriers. The team of J. Sun^22^ synthesized a TiO_2_ nanosheets/graphene 2D–2D heterostructure by introducing hydrofluoric acid (HF) to a titanate–graphene oxide (GO) mixture in a solvothermal process. The TiO_2_ nanosheets grew on and were in good contact with the GO. Zhao *et al.*^13^ and Keerthana *et al.*^15^ demonstrated the use of alkali solutions such as sodium hydroxide (NaOH) and potassium hydroxide (KOH) as a soft template in the formation of sodium dititanate (Na_2_Ti_2_O_5_) nanosheets. The mechanism involved hydrolysis of a titanate precursor, followed by a formation of the dititanate interlayers. The layers were further intercalated by the alkali ions, stabilized, and became nanosheets.^4,13,15,23^

Graphene is a superior choice for one-half of the 2D–2D heterostructure since it has a good charge transfer ability, chemical stability, and outstanding light absorption properties.^2,24^ It can be synthesized following a chemical exfoliation approach, yielding GO, which is a few layers of graphene sheet with carbon–hydrogen–oxygen functional groups. The functional groups serve as defects in the nanostructure and provide sites for the precipitation and immobilization of metal/metal oxide nanostructures. The sodium dititanate nanosheets can be synthesized and immobilized on GO *via* a hydrothermal process in an alkali solution. The 2D–2D photocatalytic heterostructure can be of great use to the photoreduction of CO_2_ to liquid fuels.

In this work, we synthesized the 2D–2D photocatalytic heterostructure of copper-doped sodium dititanate nanosheets/GO (CTGN). The heterostructure was synthesized using a one-step hydrothermal process with the addition of a NaOH soft template. Some chemical, physical and crystallographic properties of the solid samples were studied using analytical instruments, including X-ray diffraction (XRD), Raman microscope, Fourier-transform infrared spectroscopy (FTIR), UV-Visible spectroscopy (UV-Vis), high-resolution transmission electron microscope (HR-TEM), field effect scanning electron microscope (FE-SEM) and electron dispersive spectroscopy (EDS). The photocatalytic property was characterized using photoluminescence spectroscopy (PL). Liquid samples from the photoreduction of CO_2_ were analyzed using gas chromatography (GC) to obtain composition of the liquid fuels.

## 2. Materials and methods

### 2.1. Materials

All chemicals were of analytical grade and used with no further treatment. Graphite flakes (Alfa Aesar, 99.9%, -325 mesh), sodium nitrate (Fluka Chemika, 99%, NaNO_3_), potassium permanganate (Ajax FineChem, 99.0%, KMnO_4_), sodium hydroxide (Analytical reagent, Ajax FineChem, NaOH), and copper(ii) nitrate (Sigma Aldrich, Cu(NO_3_)_2_·3H_2_O) were purchased and used as received. Sulfuric acid (RCI Labscan, 98%, concentrated H_2_SO_4_), hydrochloric acid (RCI Labscan, 37%, HCl), hydrogen peroxide (Merck, 30%, H_2_O_2_), ethanol (RCI Labscan, 99.9%, C_2_H_5_OH), titanium(iv) butoxide (reagent grade, Sigma Aldrich, C_16_H_36_O_4_Ti) were used as received. The CO_2_ gas (99.9% purity) was purchased from Lor Ching Tong Oxygen (Thailand).

### 2.2. Graphene oxide (GO) synthesis

GO was chemically exfoliated by an oxidation reaction between graphite flakes and an oxidizing agent in an acid solution. The operation took place inside a fume hood and started by mixing 2 g of graphite flakes with 1 g of sodium nitrate and 50 mL sulfuric acid in a 500 mL flask. The mixture was stirred continuously and chilled in an ice-bath environment at 0 °C. During a period of 2 h, 7.3 g of potassium permanganate was slowly added to the mixture while the temperature was held below 4 °C. The container was removed from the ice bath and stirred at room conditions for another 2 h, allowing the oxidation reaction to occur. The oxidation reaction between graphite and potassium permanganate is an exothermic process, in which the heat from the reaction causes the mixture to become a thick slurry. DI water (55 mL) and hydrogen peroxide (7 mL) were added to the mixture to terminate the reaction and convert excess manganese to acid-soluble manganese oxide. The brown powder of graphene oxide (GO) was filtered from the suspension and rinsed repeatedly with 3% (v/v) hydrochloric acid solution and DI water, and dried in a vacuum oven at 60 °C for 24 h. The GO powder was further rinsed by suspending the powder in DI water using ultrasonication and a vortex mixer. The suspension was centrifuged at 9600 rpm for 15 min using the Eppendorf 5804R laboratory centrifuge machine. The supernatant was removed and replaced with fresh DI water to re-suspend the GO powder. The cycle was repeated until the pH 7 supernatant was achieved. The GO slurry was dried in a vacuum oven at 60 °C for 24 h and kept in a desiccant for future uses.

### 2.3. Synthesis of the CTGN and other control samples

To synthesize the CTGN, 0.71 mL titanium butoxide in 20 mL ethanol was mixed with 10 mL DI water. The mixture was stirred for 1 h while 3.8 mg copper nitrate (Cu(NO_3_)_2_·3H_2_O) and 10 mL sodium hydroxide solution (1 M NaOH) were added. The mixture was introduced to the 10 mL GO suspension (1.5 mg mL^−1^) and stirred for another 1 h. The mixture was transferred to a 100 mL Teflon-lined autoclave and heated at 180 °C for 8 h. The CTGN powder was obtained by centrifugation and dried in an oven at 60 °C for 24 h. The powder was further ground and kept in a desiccant. Control samples, including TiO_2_ particles (Ti), copper-doped TiO_2_ particles (CT), TiO_2_/GO (TG), and copper-doped TiO_2_/GO (CTG), were also synthesized, following a similar process. Ti was produced by adding titanium butoxide to ethanol, followed by the addition of 10 mL DI water with no soft template. The mixture was heated in a hydrothermal reactor at 180 °C for 8 h to yield the Ti particles. CT was synthesized by the hydrothermal treatment of a mixture of titanium butoxide, ethanol, DI water and copper nitrate. TG was synthesized by mixing titanium butoxide in ethanol with DI water and GO solution, followed by a hydrothermal treatment. CTG was synthesized by adding copper nitrate solution and GO solution to titanium butoxide in ethanol. The mixture was treated hydrothermally to obtain the CTG powder. Other sets of control samples, including sodium dititanate nanosheet (TiN), copper-doped sodium dititanate nanosheet (CTN) and sodium dititanate nanosheet on GO (TGN), were synthesized in presence of NaOH soft template. For TiN, a mixture of titanium butoxide, ethanol, DI water and NaOH was heated in a hydrothermal reactor. The TiN powder was obtained *via* centrifugation and dried at 60 °C for 24 h. For CTN, the hydrothermal treatment of a mixture of titanium butoxide, ethanol, DI water, copper nitrate and NaOH was utilized. For TGN, a mixture of titanium butoxide, ethanol, DI water, GO and NaOH was mixed and heated in a hydrothermal reactor.

### 2.4. Photoreduction of CO_2_ to liquid fuels

A solid powder was suspended in DI water at a concentration of 0.1 mg mL^−1^, disintegrated by ultrasonication and transferred to a 20 mL quartz photoreactor. The suspension was purged with a CO_2_ stream at a 0.3 L min^−1^ flow rate for 30 min, sealed and positioned in a closed chamber. The concentration of CO_2_ in water was expected to reach the solubility of 33 mM CO_2_ at ambient conditions.^25^ The suspension was continuously stirred during the photoreduction and was illuminated by a mercury lamp (Philips, 160 W) for 6 h. At the end of the operation, the suspension was filtrated using a syringe filter (FILTREX, PP Syringe Filter, 0.2 μm, 13 mm) to obtain a colorless liquid sample. The sample was analyzed by GC (Clarus 680; PerkinElmer) for liquid fuel composition, following the instructions of EPA method 308 with a flame ionization detector and a DB WAX column (Agilent Technologies). The GC column was preheated at 45 °C for 3 min, warmed to 70 °C for 2.5 min, and held constantly at 200 °C. The injector and detector were held at 200 °C during the operation.

### 2.5. Sample characterizations

#### 2.5.1. Light absorbance

Photocatalyst suspensions were prepared by ultrasonicating a solid powder in DI water at an initial concentration of 0.1 mg mL^−1^. The suspension was transferred to a quartz cuvette cell and introduced to 200–800 nm light from a UV-Vis spectrophotometer (UV-Vis, 1800 Shimadzu). The light absorbance data were plotted following the Tauc correlation (eqn (1)),

1 (*αhν*)^1/*n*^ = *α*_0_(*hν* − *E*_g_),

where *α*: absorption coefficient (1/*dA*), *d*: the thickness of the cell (1 cm), *A*: light absorbance, *hν*: photon energy (eV), *E*_g_: optical bandgap energy (eV), *α*_0_: constant band tailing parameter, and *n*: power factor (*n* = 2 for an indirect transition mode).

#### 2.5.2. X-ray diffraction (XRD) analysis

A solid powder was dried for 12 h in an oven at 60 °C and kept in a vacuum oven for another 12 h before XRD analysis. The powder was ground using an analytical grade mortar and pestle, sieved through a mesh to obtain the fine powder, and poured into a sample holder. The powder was analyzed by the XRD (Miniflex II, copper K-α radiation) in a 2*θ* scanning range of 5 to 80°.

#### 2.5.3. Photocurrents

The photoelectrochemical studies were conducted using the 3-electrode configuration, in which the photocatalyst was cast on a glassy carbon working electrode (WE) at a mass loading of 2.67 mg cm^−2^. The WE was employed along with the Ag/AgCl reference electrode (RE), and platinum wire counter electrode (CE) in a background medium of 0.1 M potassium chloride (KCl), and was connected to an electrochemical work station (CHI106B, CH Instrument Inc.).^16,26^ A constant potential of 0 V (*vs.* Ag/AgCl RE) was applied to the WE to provide a continual measurement of electrochemical current.

## 3. Results and discussion

### 3.1. Sample characterizations

The chemically exfoliated GO was analyzed by HR-TEM (TECNAI G2 20sTWIN, FEI), Raman spectroscopy (Horiba, XploRA PLUS with LabSpec6 software, 532 nm laser), UV-Vis (1800 Shimadzu), XRD (D8 Advance, Bruker AXS), and FTIR (FT/IR-6800, Jasco) (Fig. S1†). An image from the HR-TEM (Fig. S1(a)†) reveals the GO to be a micrometer-scale sheet with wrinkles. The Raman spectrum (Fig. S1(b)†) exhibits peaks at 1349.3 cm^−1^ and 1587.2 cm^−1^, which correspond to the disordered carbon (D band) and the graphitic carbon (G band) of the GO, respectively.^27^ The degree of disordered/graphitic carbon was presented as the *I*_D_/*I*_G_ ratio and was determined to be 0.97. This indicates that the GO sample has a high graphitic carbon structure that is comparable to the data reported in the literature.^27–29^ Fig. S1(c)† shows a UV-Vis spectrum of GO, presenting light absorption abilities in UV (200–380 nm) and visible (380–700 nm) regions. The XRD pattern of GO (Fig. S1(d)†) provides an intense peak at 12.2°, a broad peak at 25.6°, and a small peak at 43.4°. The peaks at 12.2° and 43.4° represent the (001) and (100) planes of the GO,^24,28^ while the broad peak at 25.6° marks the presence of reduced graphene oxide (rGO).^30^ The FTIR spectrum (Fig. S1(e)†) indicates the presence of hydroxyl (–OH at 3000 to 3400 cm^−1^, and 1398 cm^−1^), carbonyl (C=O at 1716 cm^−1^), alkene (C=C at 1620 cm^−1^) and alkoxy (C–O at 1054 cm^−1^).^27,30^

**Fig. 1:**
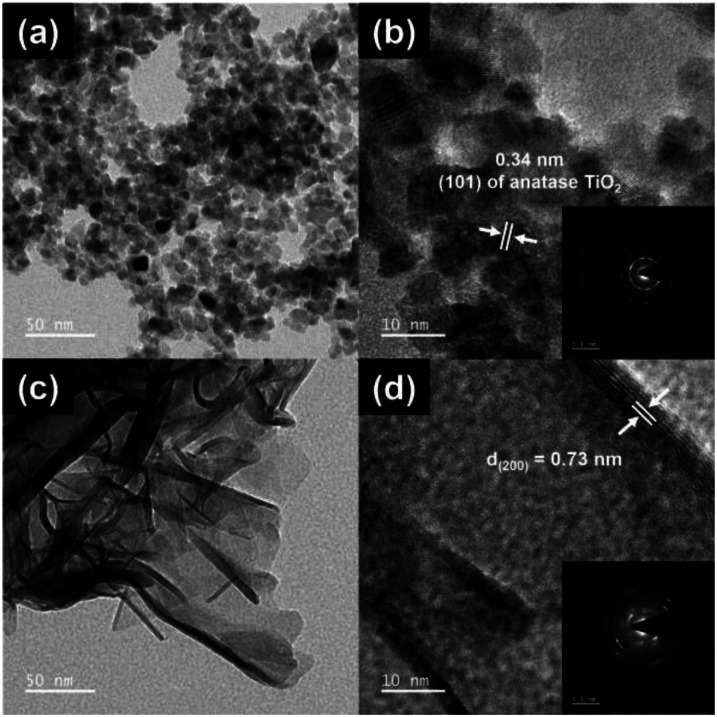
The HR-TEM and zoom-in HR-TEM images of Ti (a and b) and TiN (c and d): the light diffraction patterns of Ti (b, inset) and TiN (d, inset).

The Ti sample (Fig. 1(a) and (b)) appears to be in the form of nanoparticles with a diameter of 5.9 ± 1.5 nm while the light diffraction pattern (Fig. 1(b), inset) reveals a combination of single-crystalline and polycrystalline structures. The HR-TEM lattice fringe has a space of 0.34 nm, which correlates with the lattice pattern and the (101) planar of anatase TiO_2_.^10^ The TiN (Fig. 1(c) and (d)) has the shape of nanosheets of 77.9 ± 33.0 × 90.2 ± 37.9 nm^2^ in size.^31^ The diffraction pattern (Fig. 1(d), inset) exhibits mixed crystallography of polycrystalline and amorphous structures. Effects of the NaOH soft template on the formation of titanium nanostructure were well in agreement with the report from Zhao and his team.^13^

**Fig. 2:**
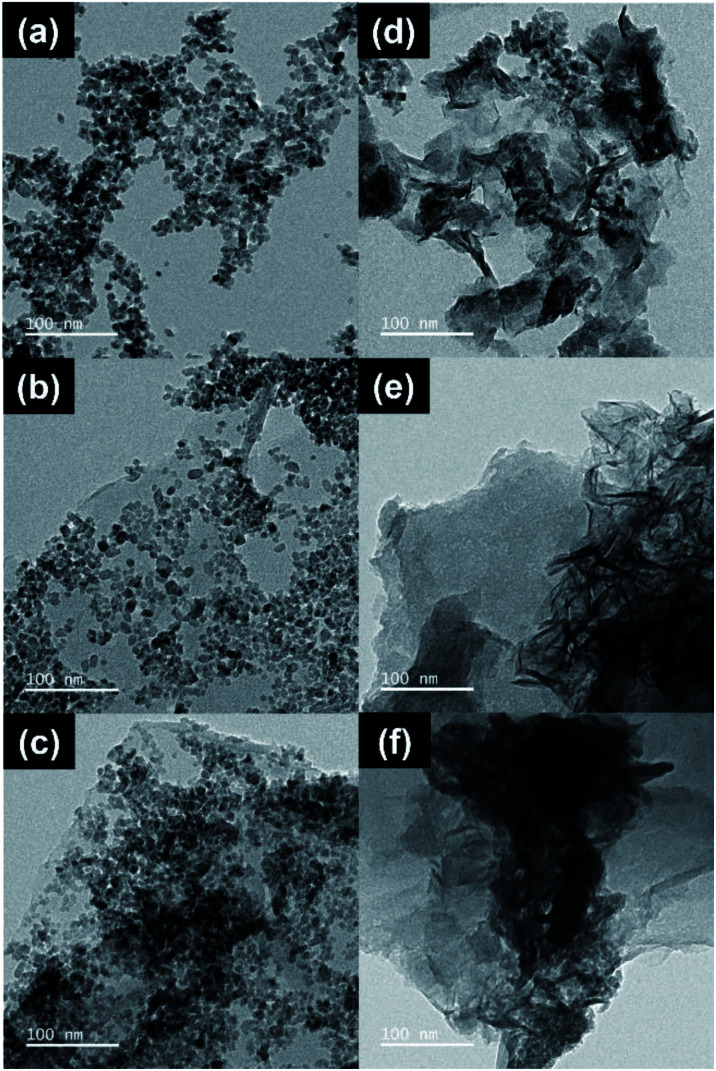
HR-TEM images showing CT (a), TG (b), CTG (c), CTN (d), TGN (e), and CTGN (f).

The HR-TEM image of CT (Fig. 2(a)) shows nanoparticles with an average size of 7.2 ± 3.7 nm. TG has 5.8 ± 1.1 nm nanoparticles immobilized on GO (Fig. 2(b)). CTG shows nanoparticles with an average size of 5.8 ± 1.1 nm (Fig. 2(c)), decorated on GO. It is clear that with no assistance from the NaOH, TiO_2_ takes the form of nanoparticles with diameter sizes ranging from 3 to 11 nm.^7,24,32^ CTN (Fig. 2(d)) appears as a combination of nanosheets and nanoparticles, in which the nanoparticle has an average size of 6.4 ± 1.8 nm and nanosheets provide an average size of 51.7 ± 18.7 × 60.1 ± 26.5 nm^2^. TGN (Fig. 2(e)) is observed to be nanoparticles and nanosheets with wrinkles, in which the nanoparticles are 5.7 ± 1.8 nm in size and nanosheets are 35.5 ± 26.0 × 83.7 ± 34.1 nm^2^. CTGN (Fig. 2(f)) shows nanoparticles and nanosheets with sizes of 6.4 ± 1.4 nm and 33.45 ± 10.1 × 86.6 ± 35 nm^2^. The atomic composition of the CTGN was analyzed using electron dispersive X-ray spectroscopy (EDS) attached to the FE-SEM (Fig. S2 and S3†). The components of copper (Cu), sodium (Na), oxygen (O), carbon (C) and titanium (Ti) were all identified.

**Fig. 3:**
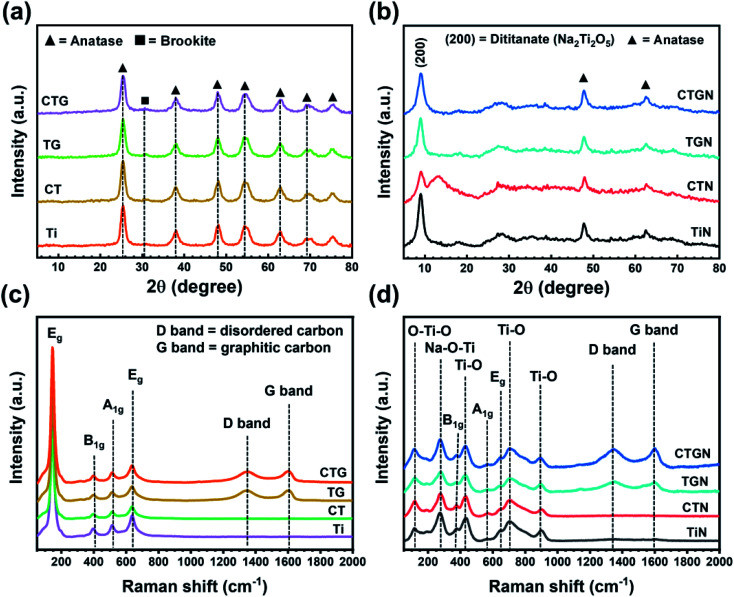
(a) XRD spectra of Ti, CT, TG, and CTG; (b) XRD spectra of TiN, CTN, TGN, and CTGN; (c) Raman spectra of Ti, CT, TG, and CTG; (d) Raman spectra of TiN, CTN, TGN, and CTGN.

Crystal structure and phase composition of solid samples were analyzed using XRD. The XRD spectra of Ti, CT, TG and CTG (Fig. 3(a)) reveal diffraction peaks at 25.3°, 38.0°, and 48.0° (2*θ*), which can be interpreted as (101), (004), and (200) lattice planes of the anatase TiO_2_.^10,18,19,23,31^ The minor peaks at 54.4°, 63.1°, 69.4°, and 75.5° can also be ascribed to anatase TiO_2_.^7,18^ A small peak at 30.6° corresponds to a weak signal of brookite TiO_2_.^10^Fig. 3(b) shows XRD spectra of TiN, CTN, TGN and CTGN with the major diffraction peak at 9.04°. The peak was indexed for the (200) plane of the dititanates.^10,23^ Other minor peaks located at 47.8° and 62.5° can be identified as anatase TiO_2_.^7,10,23^ XRD spectra from TiN, CTN, TGN and CTGN can be related to those of the sodium dititanate^15^ (Na_2_Ti_2_O_5_) and anatase TiO_2_. It is worth mentioning that no copper or copper oxide peaks were located.

The XRD results were cross-analyzed using Raman spectroscopy. For Ti and CT (Fig. 3(c)), the spectra reveal four characteristic Raman active modes for anatase TiO_2_ with E_g_ (144.6 and 636.5 cm^−1^), B_1g_ (395.6 cm^−1^) and A_1g_ (515.4 cm^−1^).^18,33,34^ For TG and CTG, the spectra peaks at 395.6, 515.4, and 636.5 cm^−1^ correspond to the B_1g_, A_1g_, and E_g_ of the anatase TiO_2_ Raman active modes. Relative intensities of the D and G band (*I*_D_/*I*_G_) for TG and CTG are determined to be 1.00 and 0.99. For TiN and CTN (Fig. 3(d)), the spectra display different modes of crystal structures in which the band signals from O–Ti–O (115.8 cm^−1^), Na–O–Ti (274.2 cm^−1^) and Ti–O (429.6, 702.4, and 893.9 cm^−1^) are indicated.^20^ Small bands observed at 377.1, 564.1, and 642.5 cm^−1^ are ascribed to the E_g_, B_1g_ and A_1g_ modes of the anatase TiO_2_. Raman active bands are in good agreement with results from the XRD analysis, revealing crystallographic structures of Ti and CTN to be a combination of dititanate (Na_2_Ti_2_O_5_) and anatase TiO_2_. For the TGN and CTGN (Fig. 3(d)), the presence of dititanate structures is noticed as the bands for O–Ti–O (112.7 cm^−1^), Na–O–Ti (274.2 cm^−1^) and Ti–O (432.7, 702.4, and 893.9 cm^−1^) are verified. The weak Raman bands for anatase TiO_2_ are spotted at 377.1, 567.1 and 642.5, which correlate to the E_g_, B_1g_ and A_1g_ of the anatase. The *I*_D_/*I*_G_ for TGN and CTGN are determined to be 0.99 and 0.99, respectively, revealing an equivalent degree of the disordered and graphitic carbon.^35,36^

**Fig. 4:**
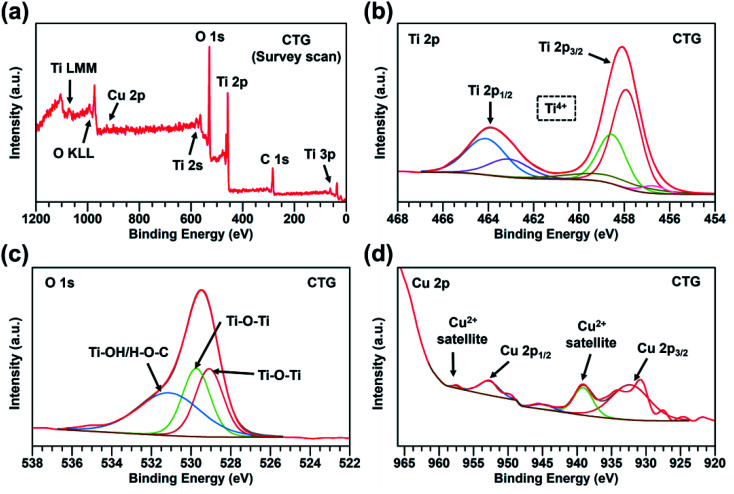
A survey scan on CTG (a); a narrow scan on CTG Ti 2p (b); a narrow scan on CTG O 1s (c); and a narrow scan on CTG Cu 2p (d).

**Fig. 5:**
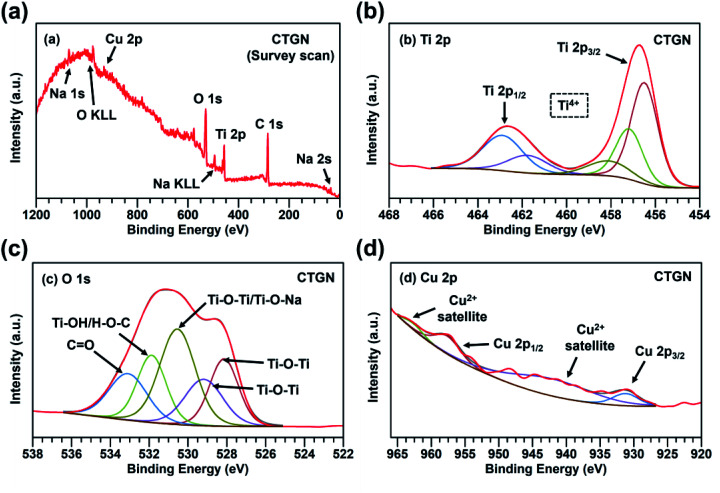
A survey scan on CTGN (a); a narrow scan on CTGN Ti 2p (b); a narrow scan on CTGN O 1s (c); a narrow scan on CTGN Cu 2p (d).

The chemical composition of the CTG and CTGN was analyzed using XPS (Fig. 4 and 5). A survey scan of CTG (Fig. 4(a)) displays elemental peaks at binding energies of 284.0, 458.0, 529.0, and 931.1 eV, which are ascribed to C 1s, Ti 2p, O 1s, and Cu 2p. The minor peaks at 36.2, 564.1 and 1073.1 eV are interpreted as Ti 3p, Ti 2s and Ti LMM. A narrow scan on the CTG Ti 2p (Fig. 4(b)) confirms the presence of Ti 2p_3/2_ and Ti 2p_1/2_ at the binding energies of 458.0 and 463.9 eV, which can be assigned to Ti^4+^. Fig. 4(c) displays a narrow scan of O 1s of the CTG sample, in which the peaks at 529.0, 529.7, and 531.1 eV can be assigned to Ti–O–Ti, Ti–O–Ti, and Ti–OH/H–O–C. A narrow scan of C 1s (Fig. S4(a)†) indicates the presence of O–C=O (288.7 eV), C–O–C (287.4 eV), C=O (285.4 eV), and C–C/C=C/C–H (284.5 eV). The C–Ti bond (283.4 eV) indicates physical/chemical interactions between TiO_2_ and GO. Oxidative states of the Cu component are analyzed in a narrow scan of Cu 2p (Fig. 4(d)), in which major peaks of Cu 2p_1/2_ (952.8 eV) and Cu 2p_3/2_ (932.4 eV) are indexed. The Cu 2p peaks verify the presence of the Cu^2+^, in which the satellite peaks at 957.6 and 939.2 eV confirm the presence of the Cu^2+^. Fig. 5 exhibits the XPS spectra of the CTGN. A wide scan of the CTGN (Fig. 5(a)) presents characteristic peaks at binding energies of 284.8, 456.8, 529.8, and 931.7 eV, which are interpreted as C 1s, Ti 2p, O 1s, and Cu 2p components. The elemental peaks of Na 2s (61.9 eV), Na KLL (494.9 eV)^37^ and Na 1s (1074.1 eV)^20^ are also observed, indicating the presence of Na and the formation of dititanate (Na_2_Ti_2_O_5_). The narrow scan of CTGN (Fig. 5(b)) shows the binding energies of 456.8 (Ti 2p_3/2_) and 462.7 (Ti 2p_1/2_) eV, which reveal the oxidative state of +4 for the Ti (Ti^4+^).^20^ A narrow scan on O 1s of the CTGN (Fig. 5(c)) displays peaks at the binding energies of 528.1, 529.1, 530.5, 531.8, and 533.1 eV, which correspond to Ti–O–Ti, Ti–O–Ti, Ti–O–Na/Ti–O–Ti,^20^ Ti–OH/H–O–C, and C=O. Carbon components of CTGN are observed in a narrow scan of C 1s (Fig. S4(b)†). Binding energy peaks of 288.2, 286.5, 284.9 and 283.4 eV are observed and can be ascribed to O–C=O, C–O–C, C–C/C=C/C–H and C–Ti components. The C–Ti interaction indicates good adhesion between the dititanate nanosheets and GO, which is the key to the formation of the 2D–2D heterostructure. Fig. 5(d) shows the Cu 2p spectrum of the CTGN, which includes Cu 2p_1/2_ (958.0 eV), Cu 2p_3/2_ (931.3 eV) and the Cu^2+^ satellite. The peaks can be analyzed as the Cu^2+^ oxidative state for the Cu on the CTGN.

**Fig. 6:**
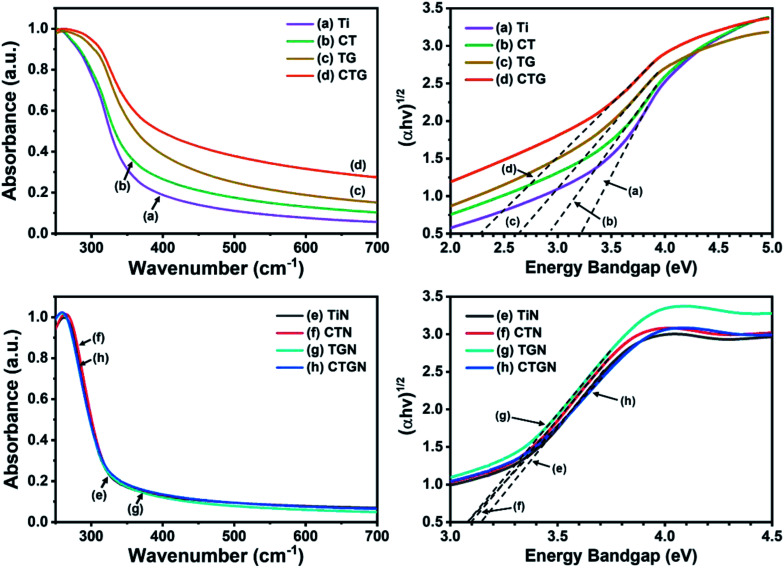
UV-Vis spectra (left) and Tauc plots (right) of Ti (a), CT (b), TG (c), CTG (d), and TiN (e), CTN (f), TGN (g) and CTGN (h).

Optical properties and energy bandgaps were characterized using UV-Vis spectroscopy (Fig. 6). The bandgap values were determined from UV-Vis spectra (Fig. 6, left) following Tauc correlation (eqn (1)) (Fig. 6, right). The Ti sample (Fig. 6(a), left) exhibits light absorption in the UV region (250–350 nm) and poor absorption in the visible range (350–700 nm). This confirms the disadvantage of normal TiO_2_ which can only absorb and be illuminated by UV light.^7,10,15^ The energy bandgap of Ti is determined to be 3.18 eV (Fig. 6(a), right), which agrees well with the reported value of 3.2 eV.^6,19^ The CT (Fig. 6(b), left) shows an enhanced light absorption ability since Cu reduces the electron–hole pairing rate and provides a transition state for photoelectrons to rest on. The bandgap for the CT sample is calculated as 2.91 eV, which is slightly lower than that of the Ti. The TG sample (Fig. 6(c), left) exhibits better light absorption ability compared to that of the Ti, absorbing UV light and part of the visible light, with the bandgap value of 2.53 eV. CTG (Fig. 6(d), left) shows light absorbance that reveals an energy bandgap of 2.28 eV as a combined effect of Cu doping and compositing of TiO_2_ with GO. For the soft template-induced samples, including TiN, CTN, TGN and CTGN, the UV-Vis spectra were also obtained (Fig. 6(e)–(h)). The TiN (Fig. 6(e)) presents a light absorption ability similar to that of the Ti, in which the TiN absorbs light effectively in the UV region but badly in the visible region. The bandgap is 3.14 eV, which is on the same scale as the reported value for the dititanate.^15^ The CTN (Fig. 6(f)) absorbs UV light and part of the visible light, showing an improvement in light absorption due to Cu doping. The energy bandgap of 3.09 eV is determined. The TGN (Fig. 6(g)) is the TiN immobilized on the GO sheet. It shows light absorption in both UV and visible regions with the bandgap value of 3.08 eV. The CTGN (Fig. 6(h)) reveals an outstanding light absorption ability from the boosting of Cu doping and GO support. Among the soft template-induced samples, the CTGN expresses the best optical properties in absorbing UV and visible light with the energy bandgap of 3.07 eV.

**Fig. 7:**
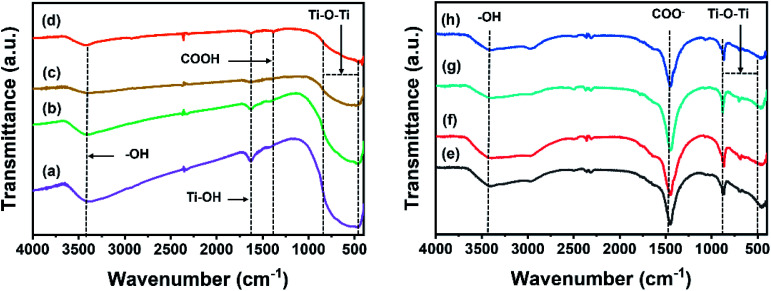
FTIR spectra of Ti (a), CT (b), TG (c), CTG (d), TiN (e), CTN (f), TGN (g), and CTGN (h).

The chemical functionality of the samples was analyzed using FTIR (Fig. 7). The IR transmittance of the Ti, CT, TG and CTG (Fig. 7, left) peaks at 1416, 1633, and 3402 cm^−1^, which correspond to the carboxyl (COOH), adsorbed water on Ti (Ti–OH),^38^ and hydroxyl (C–OH) groups.^15,27,30^ A broad peak from 500 to 800 cm^−1^ can be ascribed to the Ti–O–Ti bond.^15^ For the TiN, CTN, TGN and CTGN (Fig. 7, right), an IR transmittance peak at 3421 cm^−1^ indicates the presence of the hydroxyl group (–OH), while the peak at 1455 cm^−1^ correlates to stretching of –COO^−^ and Na^+^.^39^ A broad band between 500 to 800 cm^−1^ is related to a combined signal from Ti–O–Ti and Ti–O.^15^

### 3.2. The photoreduction of CO_2_

**Fig. 8:**
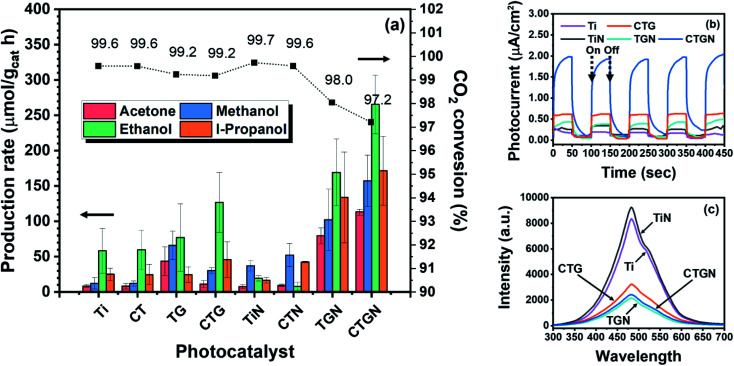
(a) Production rates of liquid fuels for Ti, CT, TG, CTG, TiN, CTN, TGN, and CTGN; (b) photocurrent responses of Ti, CTG, TiN, TGN, and CTGN; (c) photoluminescence spectra of Ti, CTG, TiN, TGN, and CTGN.

The ability to convert CO_2_ to liquid fuels is considered the most important aspect for photocatalysts. A solid powder was suspended in a CO_2_-saturated aqueous solution while illuminated by a mercury lamp for photoreduction. Four substances, including acetone, methanol, ethanol and i-propanol (iso-propanol), were detected based on GC analysis (Fig. 8(a)). For Ti, the production rates of acetone, methanol, ethanol, and i-propanol are 8, 12, 58 and 25 μmol g_cat_^−1^ h^−1^. For CT, the production rates of 8, 12, 59 and 24 μmol g_cat_^−1^ h^−1^ are determined for acetone, methanol, ethanol, and i-propanol. Photoactivity of Ti and CT are on the same scale. This can be attributed to Cu deposition, which partly contributes to the sample weight and reduces the amount of TiO_2_ in the CT. For TG, immobilizing of TiO_2_ on GO provides synergic effects which raise production rates for acetone, methanol, ethanol, and i-propanol to 42, 65, 87 and 25 μmol g_cat_^−1^ h^−1^. For CTG, the production rates of 11, 30, 125 and 45 μmol g_cat_^−1^ h^−1^ are determined for acetone, methanol, ethanol, and i-propanol, highlighting enhanced photocatalytic performance as a result of Cu doping. Photoactivity of the CTG was previously studied by our research group,^6^ in which the CO_2_ photoreduction took place in a borosilicate glass photoreactor that allowed part of the UVA and UVB but none of the UVC to penetrate. The CTG produced only ethanol (C2) at a production rate of 232 ± 98 μmol g_cat_^−1^ h^−1^, or a total carbon consumption rate of 466 μmol g_cat_^−1^ h^−1^. The total carbon consumption rate was determined considering that acetone, methanol, ethanol, and i-propanol have 3, 1, 2 and 3 carbon atoms, respectively. In this experiment, the photoreduction took place in a quartz photoreactor, and the CTG-catalyzed photoreduction yields a total carbon consumption rate of 455 μmol g_cat_^−1^ h^−1^. This indicates sufficient control over the experimental setup and addresses the point that photon energy correlated to a wavelength of light affects types of products. For TiN, the photocatalyst delivers low photoactivity, revealing only 7, 37, 19 and 16 μmol g_cat_^−1^ h^−1^ for acetone, methanol, ethanol, and i-propanol. The poor photoactivity of the TiN agrees well with the limited light absorption abilities observed in the UV-Vis spectra (Fig. 7(e)). For CTN, the photocatalyst yields production rates of 9, 52, 8 and 49 for μmol g_cat_^−1^ h^−1^ for acetone, methanol, ethanol and i-propanol. Low fuel production rates observed from TiN and CTN indicate that the dititanate is not an excellent photocatalyst on its own. For TGN, a great improvement in photoactivity is observed as the production rates increase to 79, 102, 169 and 133 μmol g_cat_^−1^ h^−1^ for acetone, methanol, ethanol, and i-propanol. For the CTGN, the highest production rates of 113, 157, 265 and 171 μmol g_cat_^−1^ h^−1^ are found for acetone, methanol, ethanol, and i-propanol. The synergic effects between dititanate and graphene contribute to the outstanding photoactivity, as obtained in cases of TGN and CTGN. The results support our hypothesis that the 2D–2D heterostructure is an excellent photocatalytic platform for CO_2_ photoreduction. The results also conform with the XPS analysis since the nanosheets and graphene adhered nicely to one another, which is an important factor for a good 2D–2D heterostructure. Moreover, the incorporation of Cu into the dititanate promotes charge separation, offering resting sites for photoelectrons to localize and detach from holes. These factors yield the best liquid production rates and the best CO_2_ photoreduction for the CTGN sample. The CO_2_ conversion in the unit of percent (Fig. 8(a)) was calculated using the eqn (2).

2 CO_2_ conversion (%) = (CO_2_ solubility − total carbon consumption)/(CO_2_ solubility) × 100

The conversions are 99.6, 99.6, 99.2, 99.2, 99.7, 99.6, 98.0 and 97.2% for Ti, CT, TG, CTG, TiN, TGN and CTGN, presenting superior photoactivity of the CTGN over other photocatalysts.

To explain the photoactivity, selected samples, including Ti, CTG, TiN, TGN and CTGN were analyzed for their photocurrents. The photocatalyst-coated glassy carbon working electrode was employed as a working electrode (WE)^16,26^ while held at a constant potential of 0 V (*vs.* Ag/AgCl RE). Photocurrents were monitored while the WE was illuminated by the UV lamp for 50 s and unlit for another 50 s to complete a cycle (Fig. 8(b)). The samples respond quickly to the incident light as the photocurrent rises and drops sharply during the illumination and the darkness. The photocurrent reveals the ability of photocatalysts to generate and transfer photoelectrons to active radicals in the medium. The intensity of the photocurrent relies on two parameters: illumination and thermal diffusion.^40^ The illumination-induced photocurrent occurs quickly in a millisecond while the thermal-induced current emits slowly within seconds. The average photocurrents for the Ti, CTG, TiN, TGN and CTGN are monitored as 1.80 × 10^−1^ ± 1.45 × 10^−2^, 6.22 × 10^−1^ ± 0.89 × 10^−2^, 2.94 × 10^−1^ ± 3.51 × 10^−2^, 4.28 × 10^−1^ ± 4.68 × 10^−2^ and 19.67 × 10^−1^ ± 5.21 × 10^−2^ μA cm^−2^. The samples took 10 to 40 seconds to reach their saturated photocurrents, indicating a combination of photo-illumination and thermal induction effects on the photocurrents. The CTGN exhibits great photoelectroactivity, providing a photocurrent 3.2 times higher than that of the CTG and comparable to that of the reported 1D nanostructure.^26^

The Ti, CTG, TiN, TGN and CTGN were analyzed further for their photoluminescence properties using photoluminescence spectroscopy (PL). The samples were excited at a wavelength of 345 nm while photons emitted from the samples in a relaxation state were collected in the 300 to 700 nm range (Fig. 8(c)). PL spectra from Ti, CTG, TiN, TGN and CTGN samples show a characteristic peak at 475 nm, indicating the main luminescence wavelength for the radiated photons. The photon emissions, in this case, correlate to the recombination effect between electrons and holes in which the higher the PL peak intensity, the higher the recombination rate.^19^ To quantify the differences in PL spectra, the quenching factor was determined^17^ by dividing the integrated area under the PL spectra over the 350 to 650 nm range of a photocatalyst with that of the TiN. The quenching factors for Ti, CTG, TiN, TGN and CTGN were determined to be 0.92, 0.53, 1.00, 0.38 and 0.42, respectively. Ti and TiN provide nearly the same value of quenching factors, indicating that the TiO_2_ (Ti) and dititanate (TiN) on their own have equivalent photoactivity. The quenching factor for CTG is significantly lower than that of Ti as a result of the copper dopant and GO support. Both the TGN and CTGN exhibit excellent characteristics for photoluminescence and photon radiation,^17,22^ showing low quenching factors. The PL supports our CO_2_ photoreduction results that the NaOH soft template-induced dititanate/graphene, TGN and CTGN, yield outstanding photoactivity.

### 3.3. Discussion of the photoreduction mechanism and photocatalytic performances

**Fig. 9:**
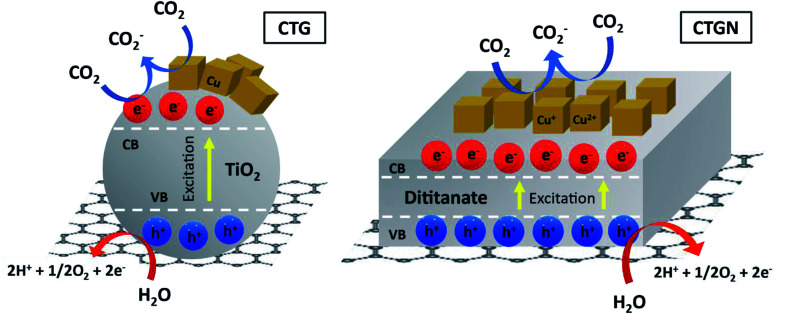
Schematic diagram showing the expected movement of the photoelectrons.

The mechanism for the photoreduction of CO_2_ was not clearly understood, as discussed in our previous work.^6^ An infinite pathway for CO_2_ conversion needs to be monitored *in situ* using high-precision spectroscopy in a controlled environment. Based on our understanding, the photo-excited photocatalyst can produce photoelectrons that couple with dissolved CO_2_. As a result, the anion carbon dioxide radical (CO_2_^−^) is generated and serves as the actual feed for the production of liquid fuel (Fig. 9). In parallel, water dissociation reaction occurs on either TiO_2_ or dititanate nanosheets, yielding protons and electrons. The total reactions, adopted from the direct electrochemical reduction of CO_2_, for the production of methanol, ethanol, i-propanol and acetone are shown in eqn (3),^41^(4),^42^(5),^43^ and (6). Several researchers reported acetone as a product of photoreduction and electroreduction of CO_2_ but have not proposed the total equation.^42,43^Eqn (6) is determined considering the number of charges involved in the redox reaction for purposes of comparison. A redox potential (*E*^0^_redox_) correlates to the tendency of a CO_2_ molecule to undergo a redox reaction and become a certain product. The reaction path with a more positive *E*^0^_redox_ value is thermodynamically favored and is more likely to occur. For the production of methanol (CH_3_OH, eqn (3)), ethanol (C_2_H_5_OH, eqn (4)) and i-propanol (C_3_H_7_OH, eqn (5)), the *E*^0^_redox_ values are −0.380, −0.329 and −0.310 V. Zhao and his team^44^ reported the *E*^0^_redox_ for an electroreduction of CO_2_ to acetone to be −0.36 V, using copper-encapsulated N-doped porous carbon as a working electrode. Based on the *E*^0^_redox_ value, the trend for the CO_2_ reduction pathway should favor i-propanol over ethanol, acetone and methanol. Our experimental results show that thermodynamics is not the only limitation and that other factors, such as the availability of photo-generated protons and electrons and mobility of charge carriers, could also contribute to the production rate.

3 CO_2_ + 6H^+^ + 6e^−^ → CH_3_OH + H_2_O

4 2CO_2_ + 12H^+^ + 12e^−^ → C_2_H_5_OH + 3H_2_O

5 3CO_2_ + 18H^+^ + 18e^−^ → C_3_H_7_OH + 5H_2_O

6 3CO_2_ + 16H^+^ + 16e^−^ → (CH_3_)_2_CO + 5H_2_O

The key to an excellent photocatalyst is to have photoelectron and proton generating sites close to one another and to inhibit electron–hole recombination (Fig. 9). The 2D–2D heterostructure tends to have outstanding properties in charge transfer and charge separation. They allow photoelectrons to travel along the shortest path through the structures and combine with other active radicals in the solution phase. The photo-generated holes can interact with and be stabilized by charges and radicals in the dititanate/GO interfaces. The interfaces can also serve as n–p heterojunctions that present resting sites for adsorption of CO_2_ and active radicals, providing sufficient CO_2_ feed and stabilizing intermediates.

The excellent photocatalytic performance of the CTGN was realized by implementing the three approaches: synthesis of the 2D dititanate nanosheets, doping of copper on the dititanate, and immobilization of the nanosheet on GO. The CTGN yields the energy bandgap of 3.07 eV, which is relatively lower than that of the Ti (3.18 eV), TiN (3.14 eV), CTN (3.09 eV) and TGN (3.08 eV), but is higher than that of the CT (2.91 eV), TG (2.53 eV) and CTG (2.28 eV). The bandgap results agree well with the literature since the dititanate provides a higher optical bandgap value when compared to that of the anatase, rutile or brookite TiO_2_. The photocatalytic performance of the CTGN was observed to be the best at CO_2_ photoreduction, producing liquid fuels at rates of 113, 157, 265 and 171 μmol g_cat_^−1^ h^−1^ for acetone, methanol, ethanol, and i-propanol. CTGN photoactivity was well supported by photoelectrochemical studies, in which the CTGN revealed a photocurrent of 19.67 × 10^−1^ ± 5.21 × 10^−2^ μA cm^−2^, which is significantly more intense than that of TiN (2.94 × 10^−1^ ± 3.51 × 10^−2^ μA cm^−2^), TGN (4.28 × 10^−1^ ± 4.68 × 10^−2^ μA cm^−2^) and CTG (6.22 × 10^−1^ ± 0.89 × 10^−2^ μA cm^−2^). The PL studies also confirm the strong photoactivity of the CTGN by revealing its low quenching factor, which can be interpreted as a low electron–hole recombination rate.

CTGN performance in photoreducing CO_2_ to liquid fuels was benchmarked with other research works (Table 1). The group of L. I. Ibarra-Rodríguez^4^ synthesized Na_2_Ti_6_O_13_/CuO/Cu_2_O heterostructure *via* a solid-state and impregnation technique. Their photocatalyst yield CO_2_ photoreduction products of formaldehyde and ethanol at a production rate of 25 and 4.6 μmol g_cat_^−1^ h^−1^, respectively. N. Lertthanaphol and his team^6^ (our previous work) utilized the one-step hydrothermal technique in synthesizing the Cu–TiO_2_/GO composite. The composite exhibited good photoactivity in reducing CO_2_ to ethanol at a production rate of 233 μmol g_cat_^−1^ h^−1^. P. Seeharaj and her team^7^ used CeO_2_/CuO/TiO_2_ heterostructure photocatalyst for CO_2_ conversion. They obtained ethanol as the only product at a production rate of 30.5 μmol g_cat_^−1^ h^−1^. H. Hsu and his team^29^ demonstrated photoactivity of the GO in photoreducing gas-phase CO_2_. The CO_2_ was continually fed in the chamber with a GO-coated disk and converted to methanol at 0.172 μmol g_cat_^−1^ h^−1^ of production rate.

**Table 1:** Summary of recent research works on the photocatalytic conversion of CO_2_ to liquid fuels

Photocatalysts	Experimental details	Bandgap (eV)	Production rate	Ref.
CTGN	Mercury lamp: 160 W; visible + UV	3.07	Acetone: 113 μmol g_cat_^−1^ h^−1^	This study
	CO_2_ in 20 mL DI water		Methanol: 157 μmol g_cat_^−1^ h^−1^	
	Catalyst: 2 mg		Ethanol: 265 μmol g_cat_^−1^ h^−1^	
	Quartz reactor: 25 mL		i-propanol: 171 μmol g_cat_^−1^ h^−1^	
Na_2_Ti_6_O_13_–5% CuO/Cu_2_O	UV-Vis lamp: 4400 μW cm^−2^; 254 nm	3.58	Formaldehyde: 25 μmol g_cat_^−1^ h^−1^	4
	2-psi pressurized CO_2_ in 200 mL DI water		Methanol: 4.6 μmol g_cat_^−1^ h^−1^	
	Catalyst: 100 mg			
	Borosilicate reactor: 250 mL			
Cu–TiO_2_/GO	Mercury lamp: 160 W; visible + UV	2.11	Ethanol: 233 μmol g_cat_^−1^ h^−1^	6 (previous study)
	CO_2_ in 25 mL DI water			
	Catalyst: 2.5 mg			
	Borosilicate reactor: 30 mL			
1% CeO_2_/3% CuO/TiO_2_	Mercury lamp: 15 W; UV	2.88	Ethanol: 30.5 μmol g_cat_^−1^ h^−1^	7
	CO_2_ in 150 mL distilled water			
	Catalyst: 150 mg			
	Borosilicate reactor with a quartz window			
Graphene oxide	Halogen lamp: 300 W	3.2–4.4	Methanol: 0.172 μmol g_cat_^−1^ h^−1^	29
	Continuous gas-flow reactor			
	Catalyst: 200 mg			
	Quartz reactor: 300 mL			

## 4. Conclusions

The copper-doped dititanate nanosheets/GO (CTGN) was synthesized following the one-step hydrothermal technique with assistance from the NaOH soft template. The CTGN showed outstanding photoactivity in photo-reducing CO_2_ to liquid fuels, including acetone, methanol, ethanol, and i-propanol at high production rates. The outstanding photoactivity of CTGN was well supported by test results from photocurrent and PL and can be attributed to the formation of 2D–2D heterostructure between the dititanate and GO. The heterostructure creates a unique interior that directs the flow of charges and reduces the electron–hole recombination rate. Such structure can provide charge carriers that assist in the photoreduction of CO_2_ and yield 2D–2D heterojunctions that accommodate active radicals and stabilize intermediates. The CTGN is demonstrated to be an outstanding photocatalyst and is considered an excellent candidate for the photoreduction of CO_2_ to liquid fuels.

## Author contributions

Napat Lertthanaphol is responsible for experimental designs, performing the experiments, data analysis, and writing of the original draft. Natthanicha Prawiset, Pornpinun Soontornapaluk and Nutkamol Kitjanukit are responsible for performing parts of the experiment, data validation and part of the experimental design. Wannisa Neamsung, Natpichan Pienusa, Kittapas Chusri, Thirawit Sornsuchat and Prowpatchara Chanthara contributed to the formal analysis of the experimental data. Poomiwat Phadungbut, Panpailin Seeharaj, and Pattaraporn Kim-Lohsoontorn contributed to funding acquisition, formal analysis and editing of the manuscript draft. Sira Srinives is in charge of writing, reviewing and editing the manuscript draft, and is the supervisor and project administrator.

## Conflicts of interest

The authors declare no financial or personal interest that affects professional judgment regarding the validity and analysis of this research.

## Supplementary Material

RA-012-D2RA04283E-s001
