# Supplementary material for: Soft template-assisted copper-doped sodium dititanate nanosheet/graphene oxide heterostructure for photoreduction of carbon dioxide to liquid fuels
Source: RSC Adv. 2022 Aug 26;12(37):24362–73. doi: 10.1039/d2ra04283e (PMC9415028; doi:10.1039/d2ra04283e)
Supplement: RA-012-D2RA04283E-s001 [file RA-012-D2RA04283E-s001.pdf]

Electronic Supplementary Material (ESI) for RSC Advances.
This journal is © The Royal Society of Chemistry 2022

Supplementary

Soft template-assisted copper-doped sodium dititanate nanosheets/graphene oxide heterostructure for photoreduction of carbon dioxide to liquid fuels

Napat Lertthanaphol[1], Natthanicha Prawiset[1], Pornpinun Soontornapaluk[1], Nutkamol Kitjanukit[1], Wannisa Neamsung[1], Natpichan Pienusa[1], Kittapas Chusri[1], Thirawit Sornsuchat[1], Prowpatchara Chanthara[1], Poomiwat Phadungbut[1], Panpailin Seeharaj[2], Pattaraporn Kim-Lohsoontorn[3], Sira Srinives[1,*]

[1] Nanocomposite Engineering Laboratory (NanoCEN), Department of Chemical Engineering, Faculty of Engineering, Mahidol University, Nakornpathom, 73170, Thailand

[2] Advanced Materials Research Unit, Department of Chemistry, Faculty of Science, King Mongkut's Institute of Technology Ladkrabang, Bangkok, 10520, Thailand

[3] Center of Excellence on Catalysis and Catalytic Reaction Engineering, Department of Chemical Engineering, Faculty of Engineering, Chulalongkorn University, Bangkok, 10330, Thailand

Keywords: Titanium dioxide, CO_2 conversion, Photoreduction, Photocatalyst, Graphene

** To whom correspondence should be addressed.*

Tel.: +66(0)84 648 2406

Email address: sira.sri@mahidol.edu (S. Srinives)

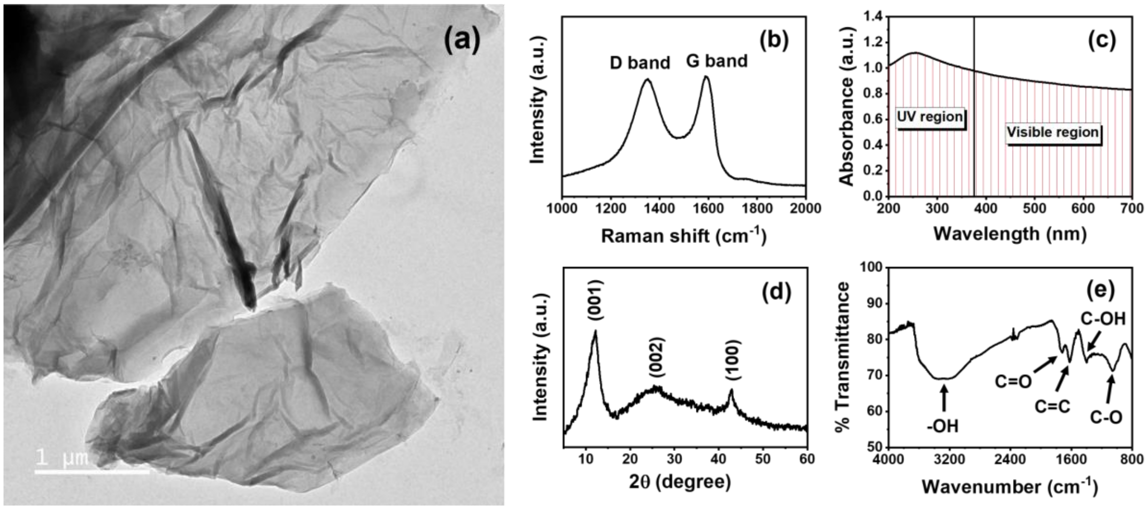

Figure S1 Characterizations of graphene oxide (GO); (a) HRTEM image, (b) Raman spectrum, (c) UV-vis spectrum, (d) XRD spectrum, (e) FTIR spectrum of GO.

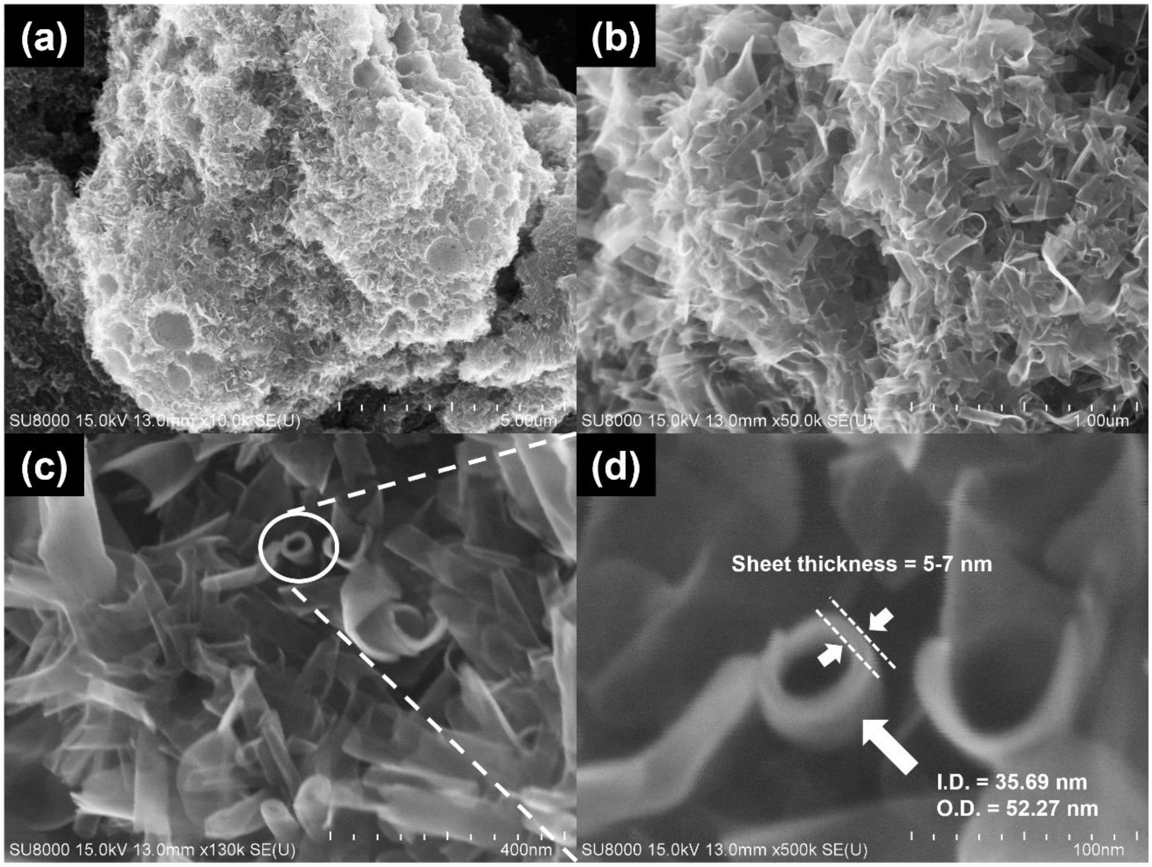

Figure S2 FESEM images showing CTGN: the composite cluster at x10k magnification(a), at x50k magnification (b), at x50k magnification (c), and rolled-TiO_2 nanosheet at x500k magnification with estimated dimension (d).

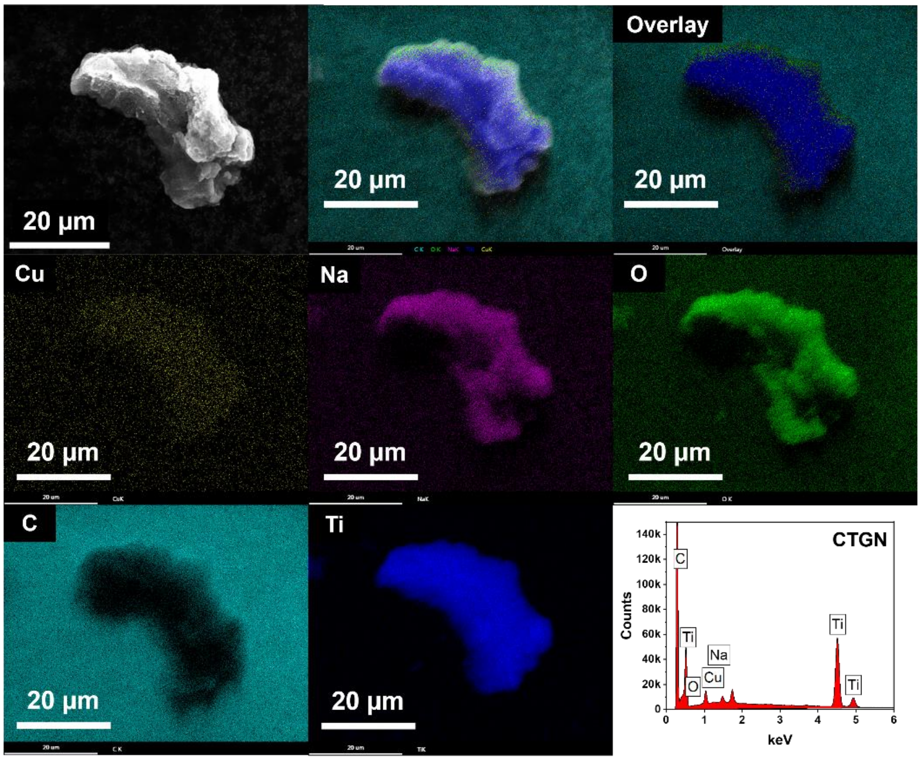

Figure S3 EDS analysis of CTGN showing an overlay of designated elements, including Cu, Na, O, C, and Ti (Titanium).

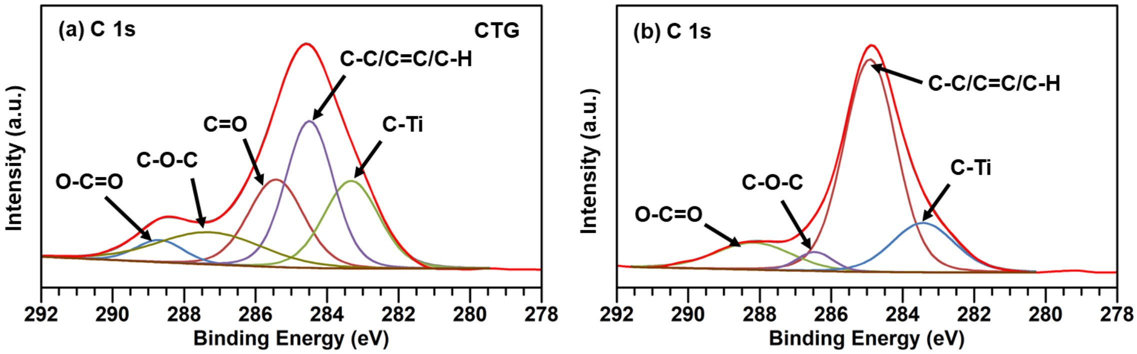

Figure S4 A narrow scan on C 1s of CTG (a) and CTGN (b)

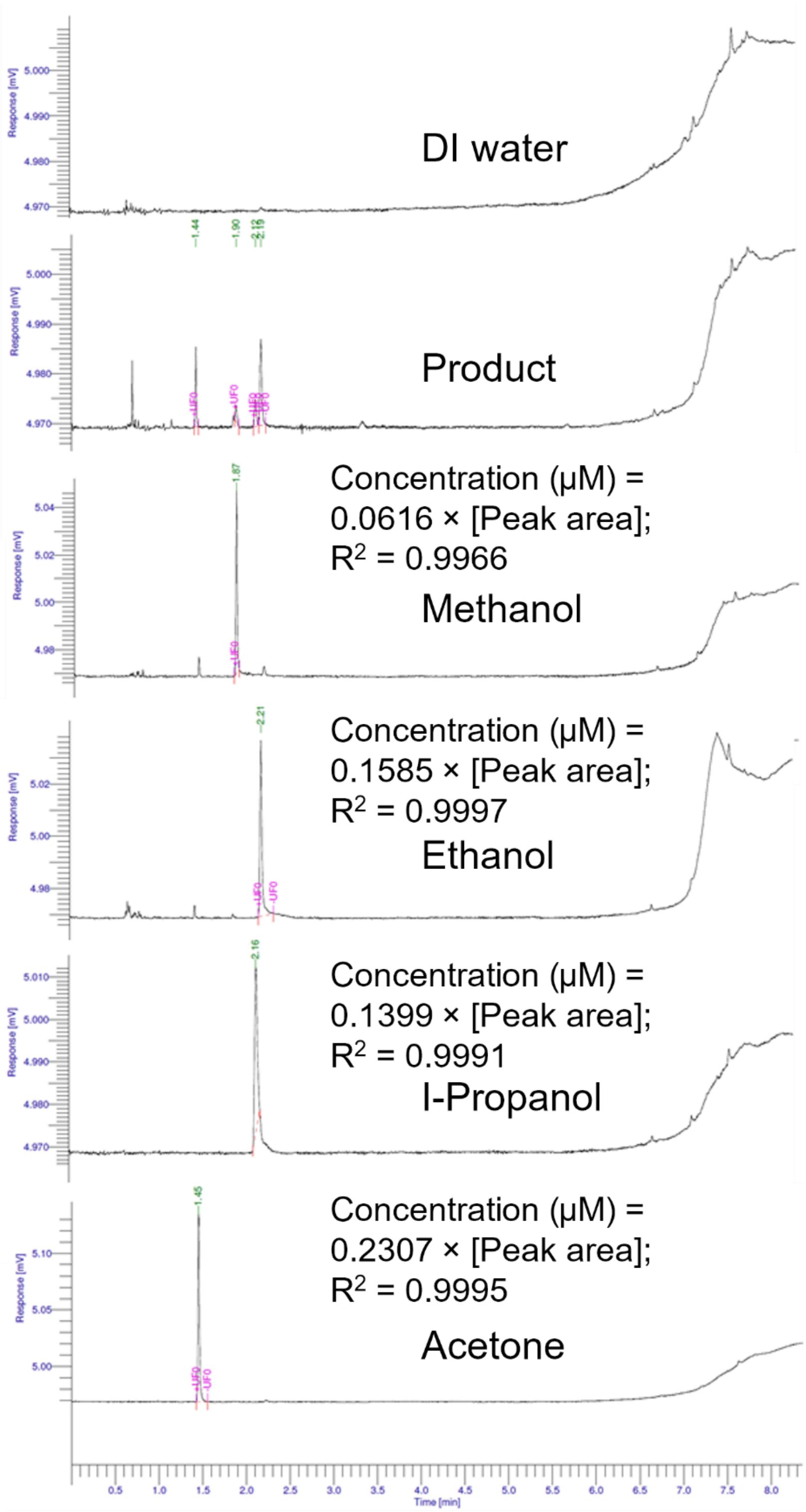

Figure S5 GC curves showing peaks from a disturbance from DI water (TOP): injection of supernatant from the CO_2 conversion experiment (2nd from the top), and a known concentration of acetone (1mM), methanol (1 mM), ethanol (1 mM), and propanol (1 mM).
